# Using a systematic review in clinical decision making: a pilot parallel, randomized controlled trial

**DOI:** 10.1186/s13012-015-0303-4

**Published:** 2015-08-15

**Authors:** Laure Perrier, Nav Persaud, Kevin E. Thorpe, Sharon E. Straus

**Affiliations:** Institute of Health Management, Policy and Evaluation, University of Toronto, 155 College Street, Toronto, ON M5T 3M6 Canada; Department of Family and Community Medicine, Li Ka Shing Knowledge Institute, University of Toronto, St. Michael’s Hospital, 30 Bond Street, Toronto, Canada; Dalla Lana, School of Public Health, Applied Health Research Centre, Keenan Research Centre, Li Ka Shing Knowledge Institute, University of Toronto, St. Michael’s Hospital, 30 Bond Street, Toronto, Canada; Faculty of Medicine, Keenan Research Centre, Li Ka Shing Knowledge Institute, University of Toronto, St. Michael’s Hospital, 30 Bond Street, Toronto, Canada

## Abstract

**Background:**

Evidence suggests that systematic reviews are used infrequently by physicians in clinical decision-making. One proposed solution is to create filtered resources so that information is validated and refined in order to be read quickly. Two shortened systematic review formats were developed to enhance their use in clinical decision-making.

**Methods:**

To prepare for a full-scale trial, we conducted a pilot study to test methods and procedures in order to refine the processes. A recruitment email was sent to physicians practicing full- or part-time in family medicine or general internal medicine. The pilot study took place in an online environment and eligible physicians were randomized to one of the systematic review formats (shortened or full-length) and instructed to read the document. Participants were asked to provide the clinical bottom line and apply the information presented to a clinical scenario. Participants’ answers were evaluated independently by two investigators against “gold standard” answers prepared by an expert panel.

**Results:**

Fifty-six clinicians completed the pilot study within a 2-month period with a response rate of 4.3 %. Agreement between investigators in assessing participants’ answers was determined by calculating a kappa statistic. Two questions were assessed separately, and a kappa statistic was calculated at 1.00 (100 % agreement) for each.

**Conclusions:**

Agreement between investigators in assessing participants’ answers is satisfactory. Although recruitment for the pilot study was completed in a reasonable time-frame, response rates were low and will require large numbers of contacts. The results indicate that conducting a full-scale trial is feasible.

**Trial registration:**

ClinicalTrials.gov NCT02414360.

**Electronic supplementary material:**

The online version of this article (doi:10.1186/s13012-015-0303-4) contains supplementary material, which is available to authorized users.

## Background and significance

Systematic reviews of randomized controlled trials are rigorous, comprehensive assessments of the evidence intended to help clinicians and other decision-makers make informed choices about health care [1]. Although there have been great advances in refining the methods of conducting and reporting systematic reviews, evidence suggests they are used infrequently by physicians in making clinical decisions [2, 3]. The lack of time to search and locate answers to questions arising in clinical practice is consistently identified as a barrier for clinicians for using evidence in practice [4–9]. Creating distilled resources that are tailored to be read quickly is one solution to delivering evidence in a manner that is convenient, portable, and timely [5, 10, 11].

Numerous tools exist that present summarized evidence-based information either from a variety of sources (e.g., clinical evidence) or specifically from systematic reviews in a shortened format (e.g., BMJ PICO abridged research articles). A recent systematic review of secondary sources (such as clinical evidence) concluded that no single online medical information tool is ideal and no single source should be relied upon to provide answers to clinical questions [12]. During the completion of a systematic review examining the impact of interventions for seeking, appraising, and applying evidence from systematic review in decision-making by clinicians or policymakers [13], we specifically screened for studies that evaluated different strategies for presenting a systematic review. Two trials were found that offered promising results but reported study quality was limited due to their small samples and participants that were drawn from a convenience sample affiliated with the Cochrane Collaboration [14, 15]. As a result, we found no studies that rigorously evaluated the impact of different formats for systematic reviews with end users.

We developed two shortened systematic review formats to enhance their use in clinical decision-making that could be offered as a companion product to a full-length systematic review. The prototypes were developed using a rigorous process including a mapping exercise, a heuristic evaluation, and a clinical content review which was followed by focus groups and usability testing with clinicians [16–18]. In order to assess these shortened formats, an online randomized controlled trial is planned to make comparisons to a full-length systematic review where the purpose of the full-scale randomized controlled trial will be to determine the impact of two distinct shortened systematic review formats compared with a traditional full-length presentation of a systematic review on generalist physicians’ understanding of evidence and their ability to apply it to a patient in a clinical scenario. The primary outcome will be the proportion of clinicians who appropriately apply the evidence from each systematic review format to the patient in the clinical scenario as measured by agreement with an expert panel’s recommendation, and the secondary outcome will be the proportion of clinicians who accurately define the “clinical bottom line” compared with that of an expert panel. Pilot studies are an important precursor to full-scale trials and other evaluations of interventions as they provide the opportunity to implement and test the context and conditions of a full randomized trial [19]. The Medical Research Council (MRC) framework provides a model to address some of the intricacies in developing and evaluating complex interventions [20]. The updated 2008 MRC framework [21] offers focused attention to early phase piloting and assessment of feasibility [21]. It identifies that large-scale evaluations can be undermined by difficulties such as acceptability, compliance, and the delivery of the intervention [21]. The piloting stage provides the opportunity to estimate rates of recruitment and retention and calculate appropriate sample sizes [21]. To prepare for the full-scale trial, we conducted a pilot study. The purpose of the pilot study is to test methods and procedures in order to refine the processes for the larger scale trial.

### Objectives

The objectives of the pilot study were to determine the feasibility of: (1) assessing participants’ answers and (2) recruiting participants in a timely manner. *A priori*, we identified that the pilot study would be considered feasible if the following were met:

1. To measure independent reviewers agreement of respondents’ answers with a kappa statistic of 0.60 or greater. Values of kappa between 0.40 and 0.59 have been considered to reflect fair agreement, between 0.60 and 0.74 to reflect good agreement and 0.75 or more to reflect excellent agreement [22].
2. To recruit a total of 54 physicians for participation within a 6-month time frame.
3. To have ≥80 % of participants who started the pilot study read the systematic review and complete the questions related to the clinical scenario within a 6-month time frame.

## Methods

### Description of pilot study

There is no formal guidance for the reporting of pilot studies for randomized controlled trials; however, Thabane and colleagues [19] provide “Recommendations for Reporting the Results of Pilot Studies” adopted from the CONSORT statement [23] that are followed for this study (Additional file 1). Two alternate systematic review formats were developed focusing on making traditional systematic reviews shorter in length, while at the same time maintaining essential information for clinical decision-making. This process is described in previous publications [16–18]. Briefly, prototypes for two formats of a shortened systematic review were developed in collaboration with a human factors engineer based on principles of user-centered design and included a mapping exercise to identify obstacles described by clinicians in using clinical evidence in decision-making, a heuristic evaluation of the prototypes, and a clinical content review of the re-formatted reviews [16]. Iterative focus groups were then conducted in order to refine the format of the prototypes, followed by usability testing to test the layout, design, and presentation during individual sessions [17, 18]. The focus groups and usability testing were completed with primary care physicians and refinements were made in an incremental process, following each round of data collection. A case study is included in the first shortened format to present contextualized information (case-based format), and the second shortened format integrates evidence and clinical expertise (evidence-expert format). This iterative development process identified component elements of the layout and placement of information resulting in templates for each of the shortened formats. As a result, templates were identified that can be used with/for individual systematic reviews to create the shortened formats.

We chose a full-length systematic review to be used for the pilot study from a list of recently published systematic reviews supplied by the Health Information Unit at McMaster University (Canada) [24] that scored 6 or better (out of 7) on the McMaster PLUS scale. These studies are rated by clinicians who are trained to assess articles they believe would be important to practicing primary care physicians. The McMaster PLUS scale is used by Health Information Unit at McMaster University to identify articles for inclusion in a secondary journal (ACP Journal Club) and BMJ Updates [24]. Initially, two physicians (one internal medicine physician and one family physician) reviewed the 89 systematic reviews supplied and independently voted on the three most relevant to generalist physicians. The final review was selected from this set of three by a third family physician independently. The following systematic review was selected for the pilot study and the shortened formats are available in Additional file 2: *Adam SS, McDuffie JR, Ortel TL, Williams JW Jr. Comparative effectiveness of warfarin and new oral anticoagulants for the management of atrial fibrillation and venous thromboembolism: a systematic review. Ann Intern Med. 2012;157(11):796-807.*

### Participants

Physicians who were in practice full- or part-time at study enrolment were recruited for participation in the pilot study by identifying family medicine and general internal medicine physicians listed in Scott’s Medical Directory [25]. This group was selected since systematic reviews summarize vast quantities of information on specialized topics which can be useful for generalist physicians. Participants had to have access to the Internet as the study was conducted online. The systematic reviews in all formats were only available in English, thus any participants who were not able to read and understand English were not eligible to participate. Anyone who had taken part in any phase of the development of the shortened versions of the systematic reviews (cased-based or evidence-expertise formats) were not eligible for recruitment.

### Procedure

A recruitment email was sent to physicians asking them to participate by clicking on the embedded link provided for the study website. Participants were sent an initial email inviting them to participate and up to three follow-up reminder emails at 1 week intervals. Clinicians were advised to have 30 to 45 min available to complete the study at the time of enrolment. Recruitment took place from July 2014 to August 2014. Ethics approval was obtained from the Research Ethics Review Boards of the University of Toronto and St. Michael’s Hospital, Toronto, Canada. An honorarium was provided to participants.

### Intervention

**Fig. 1:**
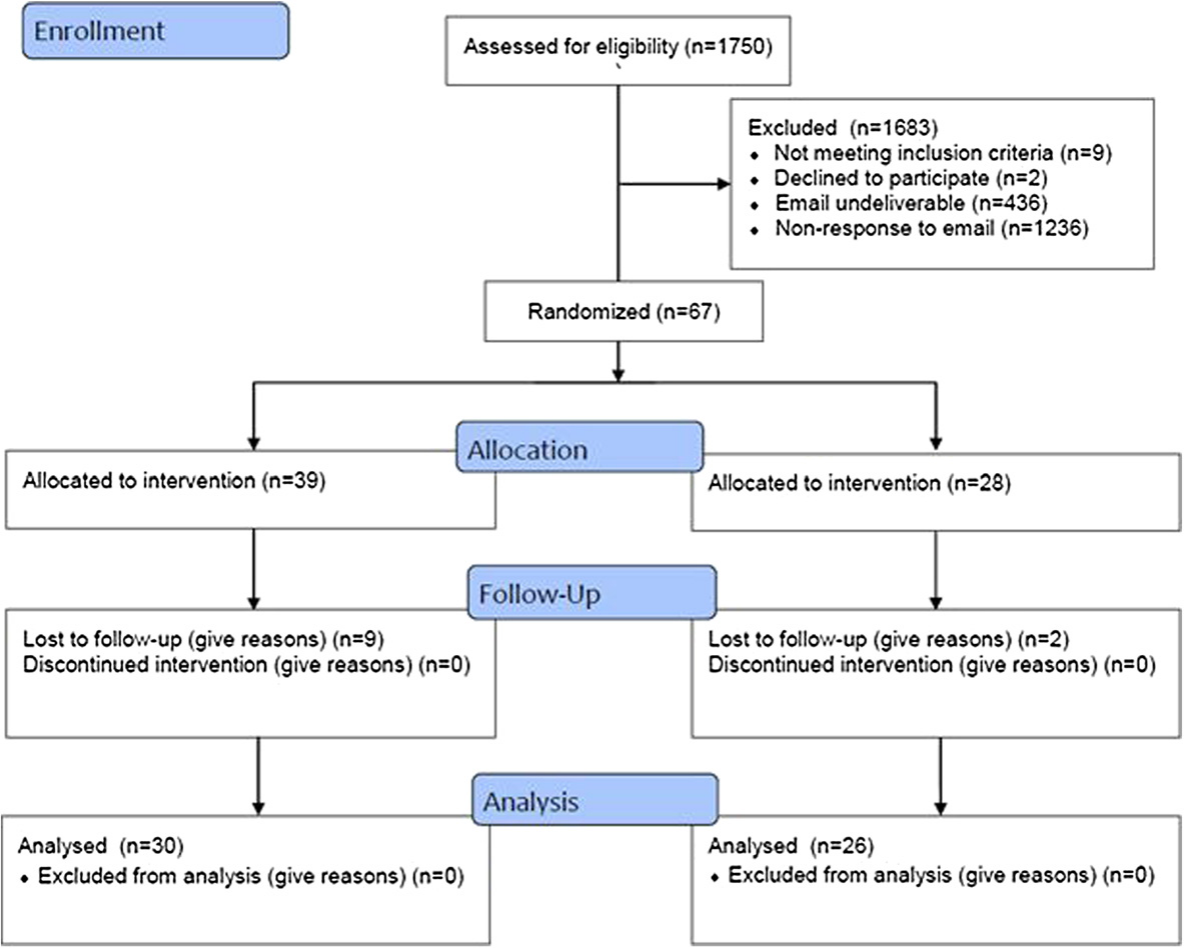
Scheme for the pilot study

The study took place in an online environment. Upon arriving at the study website, participants were asked to register for the study and complete a questionnaire that determined if they were eligible for participation. If eligible, they were directed to complete an online consent form. Participants were then randomized to one of the systematic review formats (full-length or shortened) and instructed to read the document. Participants were randomly assigned to the control (full-length systematic review) or two intervention groups (evidence-expertise or case-based shortened systematic review format) using random permuted blocks of varying sizes (Fig. 1). Twice as many participants were allocated to the control group due to the intervention having two shortened formats. Randomization was 2:1:1 and generated by a study statistician using R [26].

After reviewing the article, participants were asked to answer three questions. The first question requested that they provide the “clinical bottom line” as free text typed into an open text box. They were then instructed to read a clinical scenario (Additional file 3) relevant to the systematic review that was developed by a primary care physician and a medical educator, which provided a simulated case for analysis and decision-making. The second question asked participants if they would apply the evidence from the article to the clinical scenario (responding with “yes” or “no”). If they answered “yes,” they were asked how they would apply the evidence from the article to the clinical scenario using free text typed into an open text box. All formats were derived from the same systematic review, and all participants received the same clinical scenario.

Expert answers to the three questions were developed by a panel of three generalist physicians with expertise in evidence-based medicine and these answers served as the “gold standard” (Additional file 4). Answers provided by the participants were compared independently by two physicians (NP, SES) to the expert panel answers by two assessors who were blinded to allocation. Disagreements were resolved by discussion or a third assessor (an internal medicine physician) if agreement could not be reached.

### Sample size

To calculate the sample size for this pilot study, we used a confidence interval approach outlined by Cocks and Torgerson [27]. This method uses the full-scale trial to inform the pilot trial’s sample size, and it was calculated that 54 participants total would be required to produce an upper one-sided confidence limit that excludes 15 % when there is no difference. Sample size calculations for the full-scale trial used a small study looking at physicians’ understanding of evidence [28] and anticipated that 60 % of clinicians would be able to appropriately apply evidence from a traditional systematic review to an individual patient represented in a clinical scenario. It was expected that an increase of 15 % would be important and setting the *α* error at 0.05 (two-sided) and the *β* error at 0.15, 56 physicians were required in each group. Allowing for dropouts (assuming 20 % dropout rate), 70 physicians in each arm will be required, identifying a need for a sample size of 280 physicians for the full-scale trial.

### Analysis

#### Assessment of participants’ answers

Agreement between reviewers in independently assessing answers against the expert panel answers will be determined by calculating a kappa statistic.

The following data was collected to provide a robust appraisal of factors related to evaluating answers provided by participants that inform time, resource, and management issues:

- ▪ The initial amount of time taken to assess participants’ answers (i.e., before resolving disagreements)
- ▪ Data management (e.g., challenges experienced by study personnel, reported ad hoc)
- ▪ Data completeness (i.e., were any important data forgotten about or not provided)
- ▪ Data collection tools (i.e., do participants provide multiple answers or unanticipated answers to study questions)

#### Rate of recruitment

The rate of recruitment for the pilot study was assessed by reporting the following:

- ▪ The amount of time required to recruit participants by email
- ▪ Completion rate (i.e., the proportion of participants finishing all questions in the study divided by the number of participants starting the study)
- ▪ Contact rate (i.e., the proportion of all cases in which contact was made with a possible participant)
- ▪ Cooperation rate (i.e., the proportion of participants completing the study out of all contacts)
- ▪ Response rate (i.e., contact rate multiplied by cooperation rate)
- ▪ Traceable rate (i.e., all possible participants that can be located)
- ▪ Eligibility criteria (i.e., how many people reported they do not meet eligibility criteria)
- ▪ Time for participants to complete the study (i.e., from the time the eligibility page loaded, which is the first page after they login, until the “thank you” webpage during a single continuous session)
- ▪ Time for participants to complete the trial was calculated as the time the eligibility webpage loaded (i.e., the first page after they login) until the time the “thank you” webpage loaded that signalled the end of the session.

## Results

Recruitment emails were sent to 1750 potential participants and 436 of those emails were undeliverable. Sixty seven participants were randomly allocated to the intervention or control group, eleven people partially completed the study, and two people refused to participate. No reply was received from 1236 potential respondents and nine identified themselves as ineligible. Fifty six participants completed the pilot study.

Twenty-two females and thirty four males completed the pilot study (Table 1). One participant was identified as an internist (general internal medicine) and 55 as primary care physicians. Twenty-two participants had practiced medicine for 16–25 years, eighteen had more than 25 years of experience, nine had 11–15 years, six had 5–10 years, and one practiced for less than 5 years. Forty-three participants worked in private offices or clinics and seventeen in community hospitals. Nine physicians reported practicing in an academic health science centre, eight in a nursing home/home for the aged, and six in a community clinic/community health centre. Three participants worked in free standing walk-in clinics and five listed other places including home care, detox centre, and home-based palliative care. Most participants (39 out of 56) listed their practice population as urban/suburban. Seven practiced in a small town, six in a rural setting, and four in an inner city. Twenty eight (out of 56) respondents indicated they had not participated in critical appraisal training, and 54 (out of 56) stated they had no experience with conducting or publishing systematic reviews.

**Table 1:** Demographics and work profile of study participants

	Participants (*n* = 56)
Sex	
Women	22
Men	34
Area of practice	55
Primary care physician	1
Internist (general internal medicine)	
Years in practice	
<5 years	1
5–10 years	6
11–15 years	9
16–25 years	22
>25 years	18
Work setting^a^	
Private office/clinic (excluding free standing walk-in clinics)	43
Community clinic/community health centre	6
Free-standing walk-in clinic	3
Academic health sciences centre	9
Community hospital	17
Nursing home/home for aged	8
Others ((1) Home care, (2) detox centre/hospice home, (3) home-based palliative care, (4) home visits, and (5) community-based academic teaching clinic)	5
Practice population	
Inner city	4
Urban/suburban	39
Small town	7
Rural	6

^a^Participants listed all settings where they worked (up to five settings per person)

### Assessment of participants’ answers

Agreement amongst assessors was determined by calculating a kappa statistic. This was calculated at 1.00 (100 % agreement) reflecting excellent agreement for question 1 (identify the “clinical bottom line”). A kappa statistic for question 3 (apply the evidence from the systematic review to the clinical scenario provided) was calculated at 1.00 (100 % agreement) reflecting excellent agreement. Participants were given the choice as to whether they felt they could apply evidence from the article they had read to the clinical scenario and five participants indicated they could not. As a result, question 3 had 51 answers. Consensus was achieved by reviewing original answers and discussion.

The other factors related to evaluating answers provided by participants were the time to assess answers, data management, data completeness, and data collection tools. The mean time to assess participants’ answers was 38 s (SD 40.45). The online study environment for the assessment of participant’s answers provided data management challenges. Initially, answers from all participants appeared in one long sequence with a “Save” button at the bottom to be clicked at the end once all assessments had been completed. Although this format performed well during testing, the first time it was used during the pilot study, it failed to save the assessors decisions. This area was re-developed so that the assessment of each individual participant’s answers could be saved before moving on to the next submission. With regard to data completeness, one assessor indicated that participant’s often failed to consider all the clinical information in the clinical scenario provided. This raised the question of whether the clinical scenario appeared for participants to refer to when answering both question 2 (would they apply the evidence from the article to the clinical scenario) and question 3 (apply the evidence from the systematic review to the clinical scenario provided). Upon review, it was found that the clinical scenario did not appear on the webpage with question 3 (apply the evidence from the systematic review to the clinical scenario provided). Also, participants were not approaching question 3 (apply the evidence from the systematic review to the clinical scenario provided) in the comprehensive manner that was anticipated, and a minimum criteria was implemented as a scoring approach for this question. Concerning feedback related to data collection tools, one participant emailed to say, “I’m so sorry I only put one sentence in the first question—it looked like a small box so I thought you were looking for a one line summary”. Further, 25 participants were locked out of the system due to inactivity and when contacted, they agreed to complete the study. One participant specifically asked if they would be able to continue where they had stopped but the online system had been constructed so that the study would have to be started over again upon returning to the study website. It was decided to revise the system so that participants could return to the study at the point where they had previously stopped.

### Rate of recruitment

The first recruitment emails were sent out on 6 July 2014, and the last participant completed the study on 27 August 2014 thus taking 56 days to recruit 56 participants. Eighty-three percent of participants that started the pilot study read the systematic review and completed the questions related to the clinical scenario. The response rate for the pilot study was 4.3 %. The contact rate was 6.0 %, and the cooperation rate was 71.7 %. The traceable rate (i.e., all possible participants that can be located) was calculated at 75.1 %. Formulae and results for calculating outcome measures are shown in Additional file 5. Nine participants reported they did not meet the eligibility criteria, and this was due to either not being a primary care physician (six participants) or being retired from practice (three participants). The mean time for participants to complete the study was 22 min and 31 s (SD 19 min, 59 s). The time to completion was determined using only the respondents that were able to finish the study in one uninterrupted session without being locked out of the online study environment (31 out of 56 participants). Participants were locked out of the online study environment after 3 h of inactivity and once this happened, they were contacted in order to be given the opportunity to complete the study (25 participants). The participants that were locked out (but finished the study) were not used to calculate the mean time to complete the study. The 3 h of inactivity would have falsely inflated the times since respondents indicated they were locked out due to being called away to complete another task, not due to needing this amount of time to complete the study. Table 2 identifies all modifications made to the study.

**Table 2:** Modifications to the pilot study

	Modifications
To be completed	▪ Recruitment email: change to indicate study takes an average of 25 min to complete
	▪ Increase size of text box (for participants’ answers) to question 1
	▪ Addition of clinical scenario to webpage presenting question 3
	▪ Revision of scoring approach
Completed	▪ Re-design of how assessors save their responses
	▪ Allow participants to return study at the point where they had been locked out

## Discussion

Fifty-six participants were recruited within a 6-month period, and we had a response rate of 4.3 %. Eighty-three percent of participants that started the pilot study completed all questions in the study. For our primary outcome, a kappa statistic of 1.00 reflecting excellent agreement was calculated for question 1 (identify the “clinical bottom line”) and 1.00 for question 3 (apply the evidence from the systematic review to the clinical scenario provided). We reached the three specific goals that had been set of agreement between investigators assessing answers to a clinical scenario with a kappa statistic greater than 0.60, recruiting 54 physicians, and having ≥80 % of participants complete the pilot study once they started within a 6-month time frame.

The response rate for the pilot study is low at 4.3 % and suggests that sources and recruitment strategies other than Scott’s Directory should be considered such as partnering with a physician professional association or general medicine journal; however, low response rates are characteristic of studies recruiting physicians as participants [29]. All feasible strategies were used to improve the response rate including offering an honorarium and repeated contact [29]. Numerous contacts will be required in order to obtain a sample for the full trial so that it is powered appropriately to assess statistical significance. The mean time for participants to complete the trial was less than 25 min for a single session on average and is less than the 30–45 min originally anticipated. This allows for more accurate information to be conveyed to participants during recruitment and hopefully will facilitate recruitment. An additional incentive to consider is to provide continuing medical education credits.

The pilot study provided the forum to find out how much information participants would provide and compare it to the expert panel answers in order to refine this process. Assessors revised the scoring approach and established a minimum criterion for evaluating answers. The time to evaluate answers was calculated at 38 s per participant and is a reasonable amount of time that does not over-burden assessors.

The full set of data collected with regard to evaluating answers provided by participants informed management issues with regard to the online study environment and contributed to the purpose of testing methods and procedures in order to refine the processes for the larger scale trial. First, revisions were made to the website including altering the re-design of how assessors saved their responses and allowing participants who had been locked out to return to the study at the point where they had left off so they did not lose any of their previous entries. Given that lack of time is identified as contributing to the difficulties with recruiting physicians [29], this was a sensible alteration to the study. It also reinforced the necessity of the pilot study to provide the opportunity to rectify these problems before commencing with the full-scale trial. Second, participants’ answers did not match the extensiveness of the expert panel answer for question 3 (apply the evidence from the systematic review to the clinical scenario provided). Two issues may have influenced this in that there was no accurate manner in which to describe to participants how much or little to provide in their answer. Also, the clinical scenario was not listed alongside question 3 and relying on memory may have contributed to participants not providing comprehensive answers, especially for those returning to the study environment after being locked out. The clinical scenario had multiple components that needed to be considered in order to answer question 3 (apply the evidence from the systematic review to the clinical scenario provided), and adding the clinical scenario to this webpage is a modification that will be necessary before launching the full-scale trial. Finally, for participants that were locked out of the system due to more than 3 h of inactivity, all indicated that this was because they had been called away to another task and not due to their inability to use the online environment or understand the study. One participant sent an email indicating they had restricted their answer to question 1 (identify the “clinical bottom line”) to only one sentence due to their perception that the text box looked small. It was decided to increase the size of the text box for the full trial to guard against others making this same interpretation.

### Limitations

Several limitations must be mentioned with regard to this study. A listing was obtained from Scott’s Medical Directory for general internal medicine, however, many email addresses were invalid. The low response rate plus a small sample size used for the pilot study meant there was potential that not all groups would be represented with regard to demographics. Further, only Canadian physicians took part. Given that processes, resources, and the management of the study were being assessed, the nationality and type of physician would not impact on what is learned from the pilot study. An audience that includes practitioners from more than one country, along with making contact with a larger number of internists through strategic partnerships with groups such as medical professional societies could be considered for recruitment into the full-scale trial.

## Conclusions

Prior to launching a full-scale trial, it is important to identify and assess the feasibility of process, time, and resource problems that may arise, and potential human and data management issues. This assists in understanding the expenditure of effort in order to complete the full-scale study, plus it identifies areas that require refinement. Agreement between reviewers independently assessing participants’ answers was acceptable and requires no further refinement. The time required for participants to complete the study and for assessors to evaluate answers was reasonable. As anticipated, response rates are low and recruitment will continue to require a large number of contacts in order to recruit an adequate number of participants. The testing done to the online study environment proved to uncover the majority of problems, and some changes were made during the pilot phase, with plans to implement others prior to the full-scale trial. The results indicate that conducting a full-scale trial is feasible.

## Additional files

Additional file 1:
**Pilot study checklist: items to include when reporting a pilot study. **19.2 KB Additional file 2:
**Case-based and evidence expertise shortened formats. **232 KB Additional file 3:
**Clinical scenario. **17.6 KB Additional file 4:
**Expert panel answers. **17.6 KB Additional file 5:
**Formulae for all calculations. **23.7 KB
