# Supplementary material for: Using a systematic review in clinical decision making: a pilot parallel, randomized controlled trial
Source: Implement Sci. 2015 Aug 15;10:118. doi: 10.1186/s13012-015-0303-4 (PMC4542122; doi:10.1186/s13012-015-0303-4)
Supplement: Additional file 1: — Pilot study checklist: items to include when reporting a pilot study. 19.2 KB [file 13012_2015_303_MOESM1_ESM.docx]

**Additional file 1: Pilot Study - Checklist: Items to include when reporting a pilot study**

| **PAPER SECTION** | **Item** | **Descriptor** |
| --- | --- | --- |
| TITLE *and*  ABSTRACT | 1 | Does the title or abstract indicate that the study is a “pilot"? |
| INTRODUCTION |  |  |
| Background | 2 | Scientific background for the main study and explanation of rationale for assessing feasibility through piloting |
| METHODS |  |  |
| Participants and  setting | 3 | • Eligibility criteria for participants in the pilot study (these should be the same as in the main study  – if different, state the differences)  • The settings and locations where the data were collected |
| Interventions | 4 | Provide precise details of the interventions intended for each group and how and when they were actually administered (if applicable) – state clearly if any aspects of the intervention are assessed for  feasibility |
| Objectives | 5 | • Specific scientific objectives and hypotheses for the main study  • Specific feasibility objectives |
| Outcomes | 6 | • Clearly defined primary and secondary outcome measures for the main study  • Clearly define the feasibility outcomes and how they were operationalized – these should include key elements such as recruitment rates, consent rates, completion rates, variance estimates, etc. |
| Sample size | 7 | Describe how sample size was determined  • In general for a pilot of a phase III trial, there is no need for a formal sample size calculation.  However, confidence interval approach may be used to calculate and justify the sample size based on key feasibility objective(s). |
| Feasibility Criteria | 8 | Clearly describe the criteria for assessing success of feasibility – these should be based on the feasibility objectives |
| Statistical Methods | 9 | Describe the statistical methods for the analysis of primary and secondary feasibility outcomes |
| Ethical Aspects | 10 | • State whether the study received research ethics approval  • State how informed consent was handled – given the feasibility nature of the study |
| RESULTS |  |  |
| Participant flow | 11 | Flow of participants through each stage (a flow-chart is strongly recommended).  • Describe protocol deviations from pilot study as planned, together with reasons  • State the number of exclusions at each stage and reasons for exclusions |
| Recruitment | 12 | Report the dates defining the periods of recruitment and follow-up |
| Baseline data | 13 | Report the baseline demographic and clinical characteristics of the participants |
| Outcomes and  estimation | 14 | For each primary and secondary feasibility outcome, report the point estimate of effect and its precision (e.g., 95% confidence interval [CI]) – if applicable |
| DISCUSSION |  |  |
| Interpretation | 15 | Interpretation of the results should focus on feasibility, taking into account  • the stated criteria for success of feasibility;  • study hypotheses, sources of potential bias or imprecision – given the feasibility nature of the study  • the dangers associated with multiplicity of analyses and outcomes |
| Generalizability |  | Generalizability (external validity) of the feasibility. State clearly what modifications in the design of  the main study (if any) would be necessary to make it feasible |
| Overall evidence of  feasibility | 17 | General interpretation of the results in the context of current evidence of feasibility  • Focus should be on feasibility |

*Source:* Thabane L, Ma J, Chu R, Cheng J, Ismaila A, Rios LP, Robson R, Thabane M, Giangregorio L, Goldsmith CH. A tutorial on pilot studies: the what, why and how. BMC Med Res Methodol. 2010 Jan 6;10:1.
