# Supplementary material for: Using a systematic review in clinical decision making: a pilot parallel, randomized controlled trial
Source: Implement Sci. 2015 Aug 15;10:118. doi: 10.1186/s13012-015-0303-4 (PMC4542122; doi:10.1186/s13012-015-0303-4)
Supplement: Additional file 2: — Case-based and evidence expertise shortened formats. 232 KB [file 13012_2015_303_MOESM2_ESM.pdf]

Additional file 2 [Case Based]

Warfarin

Comparative Effectiveness of Warfarin and New Oral Anticoagulants for the Management of Atrial Fibrillation and Venous Thromboembolism

Source: *Adam SS, McDuffie JR, Ortel TL, Williams JW. Ann Intern Med. 2012;157:796-807*

Conflicts of Interest: *Ortel: consultancy (Boehringer Ingelheim; Bristol-Myers-Squibb), grant (Eisai), trial participation (Pfizer; Daiichi Sankyo). Williams: grant (VA Health Services Research.)*

CLINICAL SUMMARY

EFFECTIVE

●●○ Atrial fibrillation: patients taking direct thrombin and factor Xa inhibitors had lower all-cause mortality compared with patients taking warfarin.

●●○ Risk of fatal bleeding was lower with direct thrombin and factor Xa inhibitors compared with patients taking warfarin.

●●○ Subgroup analysis suggested a higher risk of myocardial infarction with direct thrombin inhibitors than with factor Xa inhibitors.

UNKNOWN EFFECT

- Nothing to report.

UNLIKELY EFFECTIVE

- Venous thromboembolism: direct thrombin and factor Xa inhibitors did not differ for mortality, recurrent deep vein thrombosis, or pulmonary embolism compared with warfarin.

POSSIBLY INEFFECTIVE OR HARMFUL

- Nothing to report.

REVIEW INFORMATION

**Participants**	Adults, ≥18 years with history of chronic nonvalvular atrial fibrillation (AF), deep venous thromboembolism (VTE), or mechanical valve replacement (excludes pregnant population)
**Interventions**	NOAC compared to warfarin and low molecular weight heparin
**Outcomes**	Primary: thromboembolic event
**Study Types**	Randomized controlled trials (RCTs)
**Limitations**	No head-to-head comparisons of NOACs; limited data on harms

Meta-Analysis

CLINICAL CONDITION	TREATMENTS	# months follow-up in trial participants	# trials	OUTCOMES	ADVERSE EVENTS
**Atrial Fibrillation**	Direct thrombin inhibitor (DTI) / factor Xa (FXa) inhibitors compared with warfarin<br><br>Participants: 22,256 (DTI / FXa inhibitors); 22,185 (warfarin)	21–24	3	**Outcome: Death**<br>●●○<br>Favours DTI / FXa inhibitors<br>RR: 0.88; 95% CI: 0.82-0.96<br><br>**Outcome: Hemorrhagic Stroke**<br>●●○<br>Favours DTI / FXa inhibitors<br>RR: 0.48; 95% CI: 0.36-0.92<br><br>**Outcome: Hemorrhagic Stroke**<br>●●○<br>Favours DTI / FXa inhibitors<br>RR: 0.48; 95% CI: 0.36-0.92	**Fatal Bleeding**<br>●●○<br>Risk of fatal bleeding: lower with DTI / FXa inhibitors when compared with warfarin<br>RR: 0.60; 95% CI 0.46-0.77<br><br>**Major Bleeding**<br>●○○<br>Risk of fatal bleeding: lower with DTI / FXa inhibitors when compared with warfarin<br>RR: 0.60; 95% CI 0.46-0.77<br><br>**Gastrointestinal Bleeding**<br>●○○<br>Risk of gastrointestinal bleeding: increased with DTI / FXa inhibitors when compared with warfarin<br>RR: 1.30; 95% CI 0.97-1.73<br><br>**Myocardial Infarction**<br>●○○<br>Risk for myocardial infarction: no statistically significant differences between DTI / FXa inhibitors when compared with warfarin RR: 0.95; 95% CI 0.81-1.11<br><br>**Liver Dysfunction**<br>●○○<br>Risk for liver dysfunction: no statistically significant differences between DTI / FXa inhibitors when compared with warfarin<br>RR: 0.82; 95% CI 0.56-1.18
**Venous thromboembolism**	Direct thrombin inhibitor (DTI) / factor Xa (FXa) inhibitors compared with warfarin<br><br>Participants: 5417 (DTI / FXa); 5388 (warfarin)	3–12	3	**Outcome: Death**<br>●●○<br>No statistically significant difference in the effects of DTI / FXa inhibitors when compared with warfarin<br>RR: 0.97; 95% CI: 0.72-1.30<br><br>**Outcome: Recurrent Deep vein thrombosis / Pulmonary embolism**<br>●●○<br>No statistically significant difference in the effects of DTI / FXa inhibitors when compared with warfarin<br>RR: 0.95; 95% CI: 0.71-1.27<br><br>**Outcome: Thromboembolism death**<br>●●○<br>No statistically significant difference in the effects of DTI / FXa inhibitors when compared with warfarin<br>RR: 1.00; 95% CI: 0.48-2.10	

RISK RATIOS (RR)

Probability that member of exposed group will develop a disease relative to probability that member of unexposed group will develop the same disease.

RR = 1.0	*No association between risk factor and disease*
RR > 1.0	*Risk is increased*
RR < 1.0	*Risk is decreased*

STRENGTH OF RESEARCH EVIDENCE

Ratings are based on the overall quantity and quality of clinical evidence.

●●● STRONG	*Results consistent from high quality studies. Conclusions unlikely to change with further research.*
●●○ MODERATE	*Current research supports findings, however conclusions could change with further research.*
●○○ WEAK	*Limited studies available for drawing conclusions, or existing studies have significant limitations.*
