# Supplementary material for: Using a systematic review in clinical decision making: a pilot parallel, randomized controlled trial
Source: Implement Sci. 2015 Aug 15;10:118. doi: 10.1186/s13012-015-0303-4 (PMC4542122; doi:10.1186/s13012-015-0303-4)
Supplement: Additional file 3: — Clinical scenario. 17.6 KB [file 13012_2015_303_MOESM3_ESM.docx]

**Additional file 3:** **Clinical scenario**

A 76-year-old female patient with hypertension (controlled) and osteoarthritis has been having intermittent palpitations for a month, but she feels otherwise well. You order a Holter monitor which reports paroxysmal atrial fibrillation up to 100 beats. Additional investigations, including thyroid function, renal function, liver function, and complete blood count are normal. She has no history of rheumatic disease, no murmur and an echo confirms that she has no valvular problems and no heart failure. Her medications are ramipril 5mg bid, tylenol 1g tid, and occasionally some naprosyn.
