# Supplementary material for: Using a systematic review in clinical decision making: a pilot parallel, randomized controlled trial
Source: Implement Sci. 2015 Aug 15;10:118. doi: 10.1186/s13012-015-0303-4 (PMC4542122; doi:10.1186/s13012-015-0303-4)
Supplement: Additional file 4: — Expert panel answers. 17.6 KB [file 13012_2015_303_MOESM4_ESM.docx]

**Additional file 4:** **Expert panel answers**

*Question 1:* What is the bottom line of the systematic review?

*Expert Panel Answer:*

NOACs resulted in a small decrease in all-cause mortality and hemorrhagic stroke compared with warfarin in patients with AF. However, there was no difference between NOACs and warfarin in mortality and recurrent VTE in patients treated for VTE.

Despite the lack of specific antidotes for NOACs, fatal bleeding was significantly lower with FXa inhibitors than warfarin but gastrointestinal bleeding was increased with NOACs.

The frequency of myocardial infarction was higher with dabigatran than warfarin, but not FXa inhibitors. Discontinuation due to adverse effects was also higher with dabigatran.

There are no direct comparisons of patient experience and quality of life. The impact of cost needs to be considered given the expense of these drugs compared to warfarin.

Participant may mention the following issues in their answer but are not to be penalized if they do/don't:

- Gastrointestinal bleeding was increased with NOACs and there may be variable effects between and within the 2 drug classes.
- Bleeding risk appears to be increased with the use of dabigatran, age greater than 75, and in patients with impaired renal function.

*Clinical Scenario*

A 76-year-old female patient with hypertension (controlled) and osteoarthritis has been having intermittent palpitations for a month, but she feels otherwise well. You order a Holter monitor which reports paroxysmal atrial fibrillation up to 100 beats. Additional investigations, including thyroid function, renal function, liver function, and complete blood count are normal. She has no history of rheumatic disease, no murmur and an echo confirms that she has no valvular problems and no heart failure. Her medications are ramipril 5mg bid, tylenol 1g tid, and occasionally some naprosyn.

*Question 2:* Would apply the evidence from the systematic review to the Clinical Scenario

*Expert Panel Answer:* Yes

*Question 3:* Question: How would you apply the evidence from the systematic review to the clinical scenario?

*Expert Panel Answer:*

She has a diagnosis of AF and the risk of stroke is significant [participant may quantify, eg. CHAD score]. NOACs are appealing given their ease of use, lack of monitoring required and decrease in mortality and hemorrhagic stroke, albeit small [participant may quantify, eg. NNT] compared to warfarin.

Risk of gastrointestinal bleeding is greater with the use of NOACs than warfarin, and this may be exacerbated by her use of NSAIDs for osteoarthritis.

Although her age makes her eligible for provincial drug plans, NOACs may not funded by these plans [depending on the province of residence], and this expense may be unacceptable. Given all these factors, warfarin may still be the best choice for this patient especially if her INR control is good.
