# Supplementary material for: Using a systematic review in clinical decision making: a pilot parallel, randomized controlled trial
Source: Implement Sci. 2015 Aug 15;10:118. doi: 10.1186/s13012-015-0303-4 (PMC4542122; doi:10.1186/s13012-015-0303-4)
Supplement: Additional file 5: — Formulae for all calculations. 23.7 KB [file 13012_2015_303_MOESM5_ESM.docx]

**Additional file 5:** **Formulae for all calculations**

**Sample: 1750**

*Number of Participants:*

Completed study = 56

Partially completed study = 11

Refusals = 2

Other = 9 (no longer in practice; not primary care)

Non-contact = 1236

Untraceable (Undeliverable emails) = 436

**Completion Rate**

Completes

Completes + Partials

56

56+11

= 56/67

= 0.83

= 83%

**Contact Rate**

Completes + Partials + Refusals + Other

Completes + Partials + Refusals + Other + Non‐contact

56+11+2+9

56+11+2+9+1236

= 78/1314

= 0.0593

= 5.93%

**Response Rate**

Response Rate = Contact Rate x Cooperation Rate

= 0.0593 x 0.717

= 0.0425

= 4.25%

**Cooperation Rate**

Completes

Completes + Partials + Refusals + Others

56

56+11+2+9

= 0.717

= 71.7%

**Traceable Rate**

Sample - Untraceable

Sample

1750-436

1750

= 0.751

= 75.1%

*Source:* University of Waterloo. Response Rates. Available at: http://math.uwaterloo.ca/survey-research-centre/sites/ca.survey-research-centre/files/uploads/files/SRCResponseRates.pdf. Accessed August 29, 2014.
